# Postpartum Ovarian Vein and Inferior Vena Cava Thrombosis

**DOI:** 10.1155/2014/609187

**Published:** 2014-07-10

**Authors:** Harun Arslan, Sibel Ada, Sebahattin Çelik, Tayfur Toptaş

**Affiliations:** ^1^Department of Radiology, Van Training and Research Hospital, 65100 Van, Turkey; ^2^Department of Nephrology, Van Training and Research Hospital, 65100 Van, Turkey; ^3^Department of General Surgery, Van Training and Research Hospital, 65100 Van, Turkey; ^4^Department of Hematology, Van Training and Research Hospital, Kat 1, 65100 Van, Turkey

## Abstract

Postpartum ovarian vein thrombosis (POVT), which generally occurs 2–15 days postpartum, is a rare complication. It can be confused with acute appendicitis, pelvic infection, ovarian torsion, tubo-ovarian abscess, and pyelonephritis. It is associated with morbidity and mortality. Here, we present a patient with postpartum OVT and IVC diagnosed by US and CT findings. She was treated successfully with no further need for any interventional procedures.

## 1. Background

Ovarian vein thrombosis (OVT), which generally occurs between 2 and 15 days postpartum, is a rare complication. OVT occurs in right ovarian vein in almost 90% of patients. It can easily be confused with acute appendicitis, pelvic infection, ovarian torsion, tubo-ovarian abscess, and pyelonephritis. It may lead to fatal complications such as sepsis, inferior vena cava thrombosis (IVC), pulmonary emboli, and death [1–3].

Before the emergence of radiological imaging modalities, it was quite difficult to diagnose. Most of the definite diagnoses were made during the surgical interventions. But today, the features of this phenomenon are well defined by ultrasonography (US) and computerized tomography (CT). US and CT imaging are reliable and sensitive for the diagnosis of OVT [4, 5]. Here, we present a patient with postpartum OVT and IVC diagnosed by US and CT findings. She was treated successfully with no further need for any interventional procedures.

## 2. Case Presentation

A 26-year-old puerperal woman was admitted to emergency room with the complaints of severe right lower abdominal pain on the seventh day of an elective cesarean section. She had three children. Her medical and family history revealed no previous history of any thrombotic events. Her pregnancy, labor, and delivery were uneventful. Estimated blood loss during the cesarean section was 1000 cc. Prophylactic low-molecular weight heparin was not given during the postpartum period. Physical examination revealed fever of 39°C and tenderness in her right lower abdominal region. She had mild vaginal discharge.

## 3. Investigations

Complete blood count and other laboratory tests were as follows: leukocyte count, 15100/*μ*L, Hb 10.4 g/dL; MCV, 72 fL; platelets 592000/*μ*L; CRP, 3.78 mg/dL (reference range 0–0.8 mg/dL); D-Dimer, 2.571 mg/L (reference range: 0–0.5); serum creatinine 0.8 mg/dL. Urinary analysis was otherwise normal. Her body mass index was 24.2.

**Figure 1:**
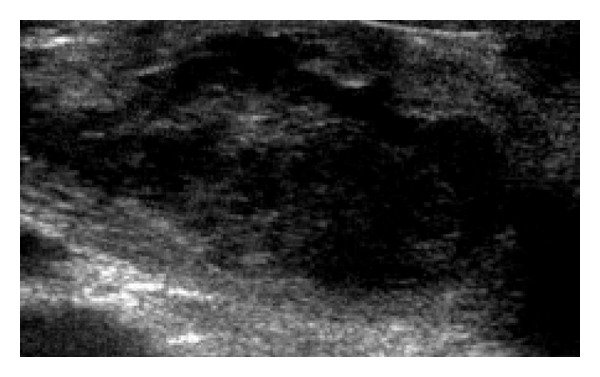
USG imaging of enlarged ovarian vein and thrombus in lumen.

**Figure 2:**
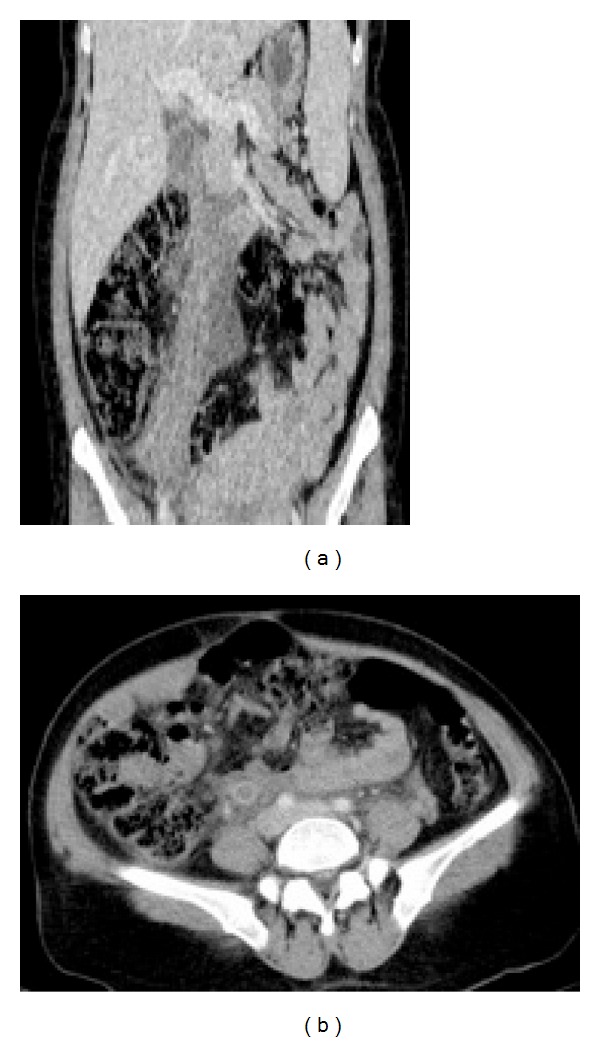
In coronal (a) and axial (b) CT images; enlarged ovarian vein, inflamed perivascular area, and thrombus in ovarian vein (OV) and inferior vena cava (IVC).

**Figure 3:**
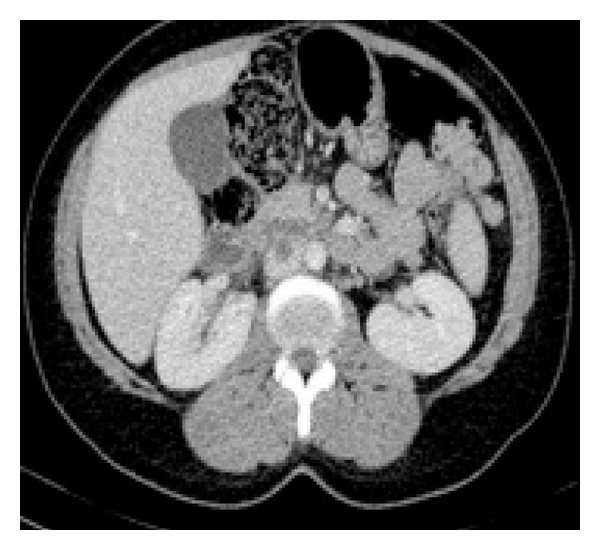
Axial CT; thrombosis in IVC.

Diagnosis of renal vein thrombosis was made due to hypoechoic areas in the right renal vein area suggesting thrombosis at first sight. However, the clinical probability of renal vein thrombosis in this woman was low. Because creatinine level was in normal ranges and she did not have hematuria, US and CT imaging were performed. There were hypoechoic areas causing filling defects within the right ovarian vein and inferior vena cava; right ovarian vein was enlarged; and perivascular edema was evident (Figures 1, 2(a), 2(b), and 3).

Laboratory investigations including protein C, protein S, antithrombin III, screening for factor Leiden V and prothrombin G20210A mutations, skin pathergy test, lupus anticoagulant, anticardiolipin antibodies, antinuclear antibody, and antidouble stranded DNA were all negative. Postpartum period was the most possible predisposing factor for the thrombosis in this woman.

## 4. Differential Diagnosis

Acute appendicitis, tubo-ovarian abscess, or endometritis was included into the differential diagnosis.

## 5. Treatment

Enoxaparin 6000 U, twice daily, and ampicillin/sulbactam 2 g, q6h, were commenced. Since there was no obvious risk factor other than puerperium, she was advised to use anticoagulation for at least 6 months.

## 6. Outcome and Follow-Up

**Figure 4:**
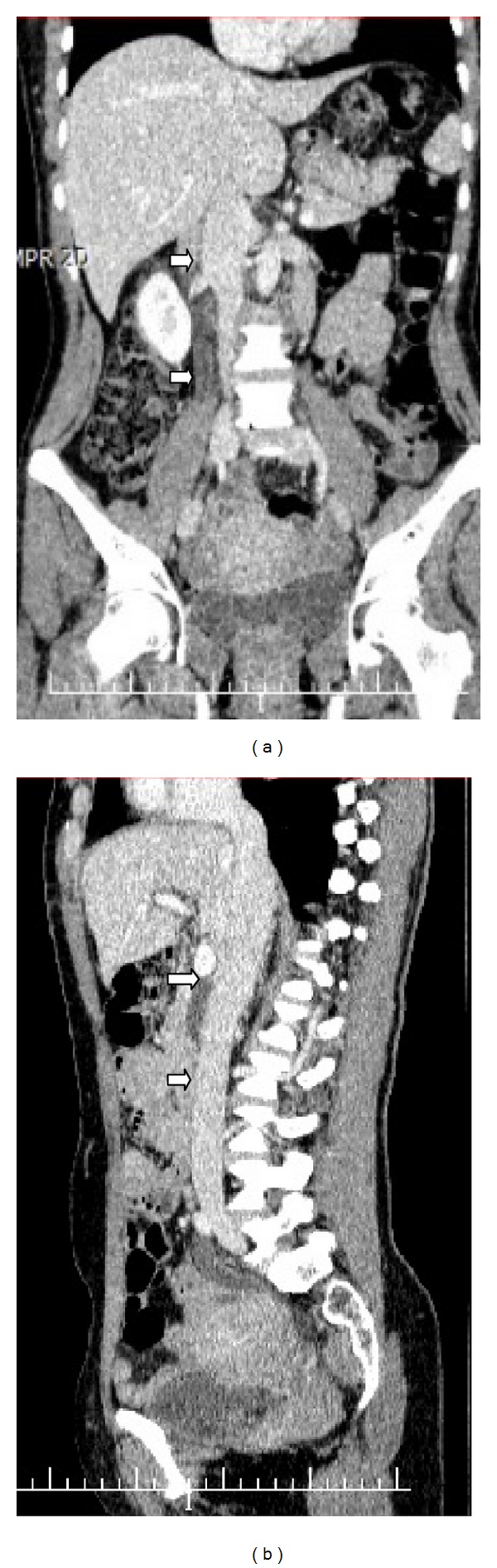
(a) Coronal and (b) sagittal images of CT with contrast enhancement revealed that OV and IVC is patent at the 12th month of OVT with no collateral circulation.

She was discharged with no complaints within a week. She received enoxaparin for 6 months and is now on follow-up free from anticoagulation with no recurrence at the 12th month of OVT (Figure 4).

## 7. Discussion

OVT complicates 1 : 500 to 1 : 2000 deliveries. In a prospective study, the incidence of OVT was 0.02% for vaginal deliveries and 0.1% for cesarean deliveries. The risk of twin versus single delivery and for cesarean section versus vaginal delivery was much higher [1].

Pregnancy-related hypercoagulability is probably the most important risk factor. Protein S and fibrinolytic activity decreases; and factors V, VII, VIII, IX, and X, platelet count, and fibrinogen increase during the postpartum period [4]. Blood flow is retrograde in left and anterograde in right ovarian vein. The physiological dextrorotation of uterus compresses on the right ovarian vein. Venous stasis develops and ovarian vein diameter nearly triples its normal size. The right ovarian vein is longer than the left one. Later also the risk of thrombosis on the right side increases [4, 6, 7]. Hypercoagulable states, such as protein S deficiency and factor Leiden V mutation, surgery, cancer and pelvic inflammatory disease, were implicated as the possible predisposing factors for OVT [8, 9]. Idiopathic OVT is rare and only limited to single case reports [10].

Clinical symptoms are not unique to OVT. Most of the patients with OVT have fever, right lower abdominal or flank pain, subtle back pain, and tenderness in groin and thigh regions. Sometimes a tender lower abdominal mass can be palpated in 50–67% of the patients. Signs and symptoms may be suggestive for acute appendicitis, urinary tract infection, pyelonephritis, adnexal torsion, puerperal endometritis, and tubo-ovarian abscess. These conditions should be included into differential diagnosis [10]. Uterine infection coexists in most of the patients either with or before the symptoms of OVT.

Ultrasound shows hypoechoic/anechoic tubular lesion in upper adnexal and inferior vena cava region. Doppler US can visualize the absence of blood flow and increased ovarian vein diameter. However, peripartum bowel and abdomen gases can reduce the imaging quality. Vascular system can be visualized better and more reliable by contrast enhanced CT and magnetic resonance imaging (MRI). CT's sensitivity and specificity are around 78%–100% and 63%–99%, respectively, in OVT. CT reveals enlarged ovarian vein, low-density appearance of venous lumen, a sharp vein wall, and perivascular edema. Thrombus in ovarian vein can extend to IVC (15%) or renal vein (12%) [11–14]. Under circumstances in which the definite diagnosis cannot be made with those modalities, it is wise to perform a diagnostic laparoscopy.

There are no specific treatment strategies implicated in the treatment of OVT. But, short-term treatment with broad-spectrum antibiotics and 3–6-month administration of parenteral/oral anticoagulants are advised [1].

The recurrence rates are comparable to that of deep vein thrombosis (~3 patient-years). Pulmonary embolism can be observed. The mortality rate was reported to be 4% in a patient series [7].

In this case report, we described a woman who experienced right OVT during the postpartum period. She presented with subtle complaints, such as fever and lower quadrant tenderness. No predisposing factors could be documented other than puerperium. She was successfully treated with one week of broad-spectrum antibiotics and low molecular weight heparin for six months. Clinicians' awareness, here, helped us for early diagnosis and successful management of a rare disease.

In conclusion, ovarian vein thrombosis is rare. It may be associated with morbidity and mortality. Early diagnosis is crucial to prevent unnecessary interventional procedures, morbidity, and mortality. OVT should be considered in differential diagnosis of a postpartum patient with the complaints of abdominal pain, fever, and leukocytosis.
